# Impact of *ARID1A* and *TP53* mutations in pediatric refractory or relapsed mature B-Cell lymphoma treated with CAR-T cell therapy

**DOI:** 10.1186/s12935-023-03122-2

**Published:** 2023-11-19

**Authors:** Yang Li, Yang Liu, Keyan Yang, Ling Jin, Jing Yang, Shuang Huang, Ying Liu, Bo Hu, Rong Liu, Wei Liu, Ansheng Liu, Qinlong Zheng, Yonghong Zhang

**Affiliations:** 1 Molecular diagnostics laboratory, Beijing GoBroad Boren Hospital, Beijing, China; 2 Department of Pediatric Lymphoma, Beijing GoBroad Boren Hospital, Beijing, China; 3 Department of Hematology/Oncology, Beijing Children’s Hospital, National Center for Children’s Health, Capital Medical University, Beijing, China; 4 Department of Hematology/Oncology, Capital institute of pediatric, Beijing, China; 5 Department of Hematology/Oncology, Zhengzhou Children’s Hospital, Zhengzhou, China; 6 Department of Hematology/Oncology, Xian Children’s Hospital, Xi’An, China

**Keywords:** Pediatric B-cell non-hodgkin Lymphomas, CAR-T cell therapies, Gene mutations

## Abstract

**Background:**

Chimeric antigen receptor (CAR)-T cell therapy has been used to treat pediatric refractory or relapsed mature B-cell non-Hodgkin lymphoma (r/r MB-NHL) with significantly improved outcomes, but a proportion of patients display no response or experience relapse after treatment. To investigate whether tumor-intrinsic somatic genetic alterations have an impact on CAR-T cell treatment, the genetic features and treatment outcomes of 89 children with MB-NHL were analyzed.

**Methods:**

89 pediatric patients treated at multiple clinical centers of the China Net Childhood Lymphoma (CNCL) were included in this study. Targeted next-generation sequencing for a panel of lymphoma-related genes was performed on tumor samples. Survival rates and relapse by genetic features and clinical factors were analyzed. Survival curves were calculated using a log-rank (Mantel-Cox) test. The Wilcox sum-rank test and Fisher’s exact test were applied to test for group differences.

**Results:**

A total of 89 driver genes with somatic mutations were identified. The most frequently mutated genes were *TP53* (66%), *ID3* (55%), and *ARID1A* (31%). The incidence of *ARID1A* mutation and co-mutation of *TP53* and *ARID1A* was high in patients with r/r MB-NHL (*P* = 0.006; *P* = 0.018, respectively). CAR-T cell treatment significantly improved survival in r/r MB-NHL patients (*P* = 0.00081), but patients with *ARID1A* or *ARID1A* and *TP53* co-mutation had poor survival compared to those without such mutations.

**Conclusion:**

These results indicate that children with MB-NHL harboring *ARID1A* or *TP53* and *ARID1A* co-mutation are insensitive to initial conventional chemotherapy and subsequent CAR-T cell treatment. Examination of *ARID1A* and *TP53* mutation status at baseline might have prognostic value, and risk-adapted or more effective therapies should be considered for patients with these high-risk genetic alterations.

**Supplementary Information:**

The online version contains supplementary material available at 10.1186/s12935-023-03122-2.

## Introduction

Mature B-cell non-Hodgkin lymphoma (MB-NHL) accounts for 50–60% of non-Hodgkin lymphomas (NHLs) in children and adolescents [1–3] and is highly aggressive, with unique epidemiology and pathological features [1]. Despite its high intensity, short-course multichemotherapy has significantly improved the cure rate of patients [4, 5]; nevertheless, some children experience induction failure, refractoriness or recurrence (r/r), and the prognosis of r/r MB-NHL is generally poor, with a cure rate of less than 30% [6–8].

Chimeric antigen receptor (CAR)-T cell therapy is an emerging therapy that has been used to treat patients with r/r lymphoma, with significant improvement in clinical outcomes and an overall response rate of 52–82% [9–11]. In our previous study, the 18-month progression-free survival (EFS) rate with sequential CAR-T cell therapy was estimated to be 78% for r/r BL [12]. Although high remission rates after CAR-T cell therapy can be achieved in patients with r/r MB-NHL, some patients still experience relapse [11, 13–15]. In recent years, much attention has been given to clinical research on factors related to CAR-T cell therapy failure. Some studies have shown that intrinsic genetic changes may affect the treatment and prognosis of patients with lymphoma [16, 17]. However, to date, the effect of the preexisting genetic landscape of MB-NHL on CAR-T cell therapy has remained unclear, and despite recent studies in DLBCL [18], few studies have been based on children.

Therefore, we selected a cohort of 89 Chinese pediatric MB-NHL patients to analyze the relationship between mutation status and genetic characteristics and CAR-T cell therapy. We sought to understand the intrinsic molecular characteristics of r/r MB-NHL patients who are not sensitive to CAR-T cell therapy in an attempt to identify molecular predictors associated with this treatment. Identifying patients at high risk of relapse who may not benefit from immunochemotherapy and/or CAR-T cell therapy can provide a clinical basis for improving clinical management and treatment strategies for these lymphoma patients.

## Patients and methods

### Patients

A total of 89 patients treated at multiple clinical centers of the China Net Childhood Lymphoma (CNCL) from February 2019 to September 2021 were included in this retrospective study. Targeted next-generation sequencing (t-NGS) with a panel of lymphoma-related genes was performed on tumor samples (diagnostic [19] and staging criteria [20] are described in supplementary materials 1.1). All patients received at least first-line chemotherapy and were evaluated and followed up after treatment [21]. There were 41 patients with initial remission and 48 patients with r/r. Among the r/r patients 40 (details of the inclusion and exclusion (I/E) criteria are shown in supplementary materials 1.2) received CAR-T cell infusion between February 2019 and September 2021 and were evaluated for responses and adverse effects (see Supplementary material 1.3, 1.4 and 1.5). The cohort included 9 patients who received CD19 CAR-T cell therapy (ClinicalTrials #: ChiCTR-1,800,014,457), 1 patient who received CD20 CAR-T cell therapy (ClinicalTrials #: ChiCTR-1,800,014,457), 22 patients who received sequential CD19-22 CAR-T cell therapy (ClinicalTrials #: ChiCTR-1,800,014,457), and 8 patients who received CD19&22 or CD20&22 CAR-T cell therapy (ClinicalTrials #: ChiCTR-2,100,045,864). The lentiviral vector was prepared by Shanghai Yake Biotechnology Co., Ltd. This study was approved by the institutional review board of Beijing GoBroad Boren Hospital, in accordance with the Declaration of Helsinki (Approval numbers: 20,210,312-KS-001Y and 20,180,114-PJ-001). Patients (or their guardians) were required to provide written consent.

### Definitions

Initial remission refers to achieving complete response (CR) after first-line chemotherapy. Relapsed or refractory (r/r) was defined as disease that was refractory (never obtaining a CR) or relapsed after first-line chemotherapy and had a partial response (PR) or no response (NR) as the best response to at least 2 cycles of salvage chemotherapy. Poor prognostic outcome was defined as meeting at least one of the following criteria: deceased, unresponsive to treatment and/or disease relapse.

### Targeted next-generation sequencing and mutational analysis

t-NGS was used to detect the mutation status of 262 driver genes related to lymphoma to identify somatic mutations, including single-nucleotide variants (SNVs) and short insertions and deletions (InDels) in the patients. First, DNA was extracted from tumor tissue samples. The genomic DNA was fragmented to approximately 200 bp by enzyme digestion, and then end repair, adaptor ligation and PCR (polymerase chain reaction) were carried out to complete prelibrary construction. A complete set of probes provided by Agilent was used to capture the sequences of 262 genes (all coding exons), and the target fragments were enriched to generate the library. A NextSeq550 sequencer (Illumina) was used for 2*150 bp sequencing analysis. The human genome hg19 was used as a reference for sequence alignment and base identification. Mutation information was annotated by the information screening function based on public databases (dbSNP, 1000 Genomes, and ESP6500). The variant allele frequency (VAF) was calculated by the number of mutants reads over the number of total reads (Supplementary 1.6).

### Statistical methods

Statistical analyses were carried out with SPSS statistics v.25 and R software v3.6.2. Actuarial survival analysis was estimated by the Kaplan–Meier method, and survival curves were generated using a log-rank (Mantel‒Cox) test. The Wilcox sum-rank test and Fisher’s exact test were applied to test for group differences. *p* values of 0.05 or below were considered statistically significant.

Overall survival (OS) was calculated from the date of the last CAR-T cell infusion to the date of last follow-up or death, and disease-free survival (DFS) was defined only for patients who achieved CR and was calculated from the date of CR after the last CAR-T cell treatment to the date of relapse or death regardless of cause. Follow-up visits for MB-NHL cases are scheduled every 3 to 6 months for the first 2 years and once every 6 months after 5 years. All patients were followed up until 30 September 2021.

## Results

### Clinical characteristics

The clinical data of the 89 patients are summarized in Table 1. The median age was 8 years old (range 0–18 years old). Of the patients, 63 had BL, accounting for most cases (73.8%), 11 (12.4%) had HGBL, 10 (11.2%) had DLBCL, and 5 had other types. This cohort predominantly consisted of males (73, 82.0%), approximately 5 times the number of females (16, 18.0%), and the sex composition was consistent with that of another study [22]. The major clinicopathological stages of the patients were stage III (43, 48.3%) and stage IV (42, 47.2%). Twenty-eight patients who achieved complete recovery or partial remission (CR) and achieved remission lasting more than 6 months after initial treatment were defined as the initial remission group (IR). Thirteen patients had CR after initial treatment, but remission lasted less than 6 months; these patients are still under follow-up. Forty-eight cases were r/r MB-NHL (r/r MB-NHL) (the groups are shown in Supplementary Table S1).

**Table 1:** Clinical data of pediatric patients with MB-NHL (n = 89)

	Level	Overall n = 89
Age (mean (SD))		8.94 (4.39)
Gender (%)	Female	16 (18.0)
	Male	73 (82.0)
Disease subtype (%)	BL	63 (70.8)
	HGBL	11 (12.4)
	DLBCL	10 (11.2)
	Others	5 (5.6)
Stage (%)	II	2 (2.2)
	III	43 (48.3)
	IV	42 (47.2)
	Unknown	2 (2.2)
CNS (%)	CNS1	52 (58.4)
	CNS2	15 (16.9)
	CNS3	20 (22.5)
	Unknown	2 (2.2)
Group (%)	r/r	48 (53.9)
	initial remission (≥ 6mounths)	28 (31.5)
	initial remission (<6mounths)	13 (14.6)
CAR-T or not	No	49 (55.1)
	Yes	40 (44.9)
CAR-T Response (n = 40)	NR	15 (37.5)
	R	25 (62.5)

BL, Burkitt Lymphoma; HGBL, High-grade B cell Lymphoma; DLBCL, Diffuse Large B Cell Lymphoma; CNS,Central Nervous System; NR, no response; R, response

The median follow-up was 11.4 months (95% CI: 9.9–13.5 months). The one-year OS of r/r MB-NHL was 43.5% (95% CI, 29.4–64.3).

### The mutation landscape of MB-NHL in children

The mutational profiles of 89 MB-NHL patients are presented in Supplemental Figure S1. We detected 324 somatic driver mutations involving 89 genes that have been reported in lymphomas with an impact on treatment decisions, diagnosis, or prognosis. On average, each patient carried 5 mutations, ranging between 1 and 13. Among patients with each mutation, 73 harbored at least three variants (82.0%), and 4 of them carried ≥ 10 mutations; one patient carried 13 mutations, the highest number detected.

The most commonly mutated genes were *TP53* (in 59 of 89 patients; 66%), followed by *ID3* (in 49 patients; 55%), *ARID1A* (in 28 patients; 31%), *CCND3* (in 26 patients; 29%), *DDX3X* (in 23 patients; 26%), and *GNA13* (in 14 patients; 16%) (Supplementary Fig. S1). The mutated genes are mainly related to the *TP53* signaling pathway, chromatin remodeling, the cell cycle, epigenetics and the NF-κβ signaling pathway.

### Differences between IR MB-NHL and r/r MB-NHL patients

**Fig. 1:**
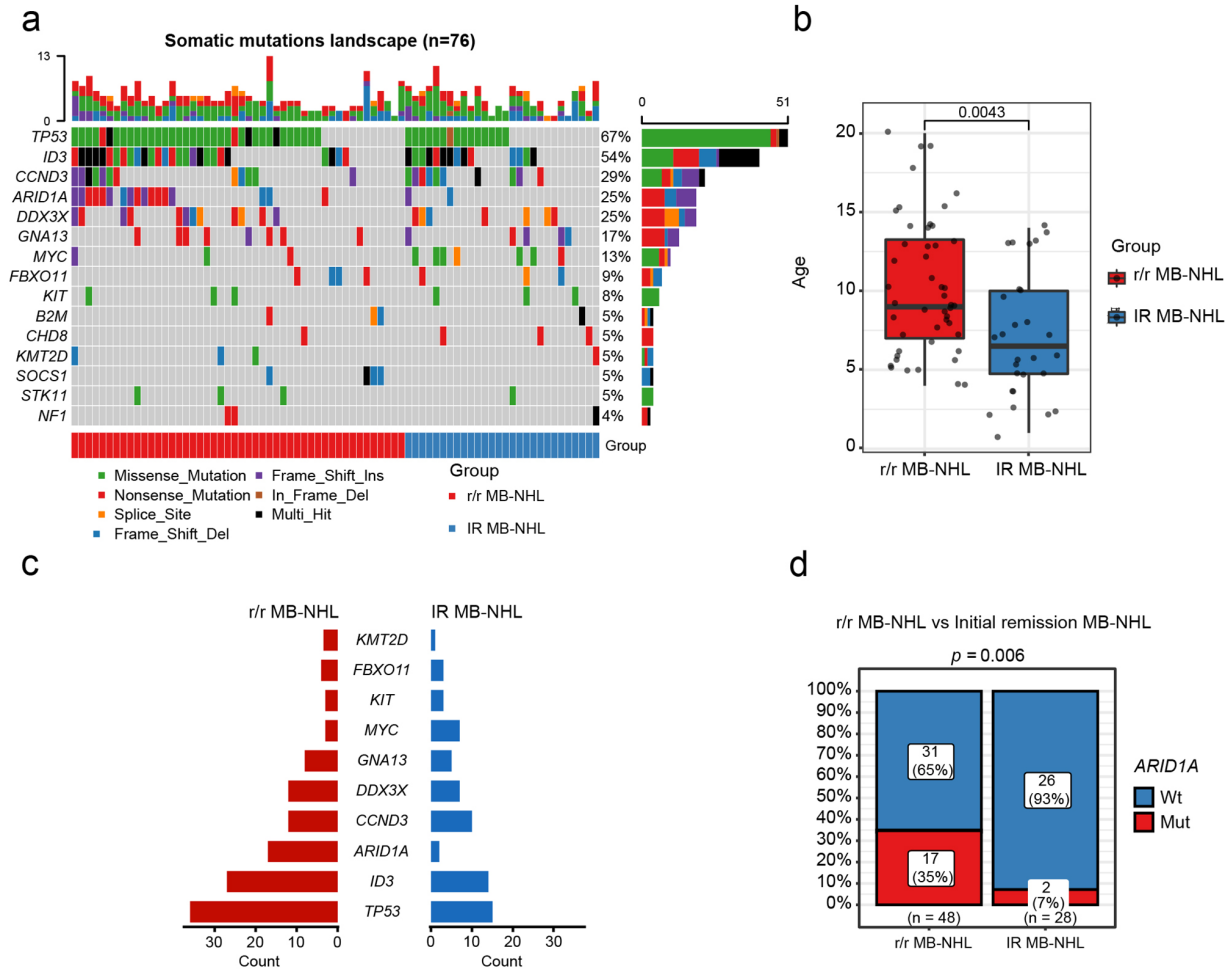
Comparison between r/r MB-NHL and initial remission patients. (**a**). The mutational spectrum of r/r MB-NHL (n = 48; left) and initial remission patients (n = 28; right). Overview of the top 30 mutated genes with different forms of mutation and their frequencies. Side bar plots indicate the incidence of mutations in a gene in 76 patients. Top bar plots indicate the number of mutated genes per participant. (**b**). The box plot indicates a comparison of ages between r/r MB-NHL (left, red) and initial remission patients (right, blue). (**c**). Comparison of mutation profiles between r/r MB-NHL (left, red) and initial remission patients (right, blue). (**d**). Comparison of the incidence of *ARID1A* mutations in r/r MB-NHL (left) and initial remission patients (right). Wt, wild-type *ARID1A*; Mut, *ARID1A* mutation

By comparing the clinical characteristics of the patients with IR MB-NHL (n = 28) and those with r/r MB-NHL (n = 48), it was found that the age of the latter was significantly higher than that of the former (IR MB-NHL: median = 6.5 years, IQR: 4.25-10 years; r/r MB-NHL: median = 9 years, IQR: 7-13.75, *P* = 0.00043) (Fig. 1b and Supplementary Table S2). There are few studies on the correlation between age and prognosis in pediatric lymphoma, but our results are supported by evidence showing that pediatric NHL patients have a significantly better prognosis than adult patients with the same histological subtype [23]. There were no significant differences in clinicopathological stage, sex or CNS between the two groups (not shown).

In 28 IR MB-NHL patients, the most commonly mutated genes were *TP53* (54%, 15/28), *ID3* (50%, 14/28), *CCND3* (36%, 10/28), *DDX3X* (25%, 7/28), *MYC* (25%, 7/28), and *GNA13* (18%, 5/28). In 48 r/r patients, the most commonly mutated gene was *TP53* (75%, 36/48), followed by *ID3* (56%, 27/48), *ARID1A* (35%, 17/48), *CCND3* (25%, 12/48), *DDX3X* (25%, 12/48), and *GNA13* (17%, 8/48). Compared with MB-NHL, *TP53* (75% vs. 54%) and *ARID1A* (35% vs. 7%) showed a higher incidence of mutation than IR MB-NHL (Fig. 1a and c), with the incidence of *ARID1A* mutation reaching a significant difference (*P* = 0.006, Fig. 1d). The results suggest that *ARID1A* mutations are enriched in resistant disease.

**Fig. 2:**
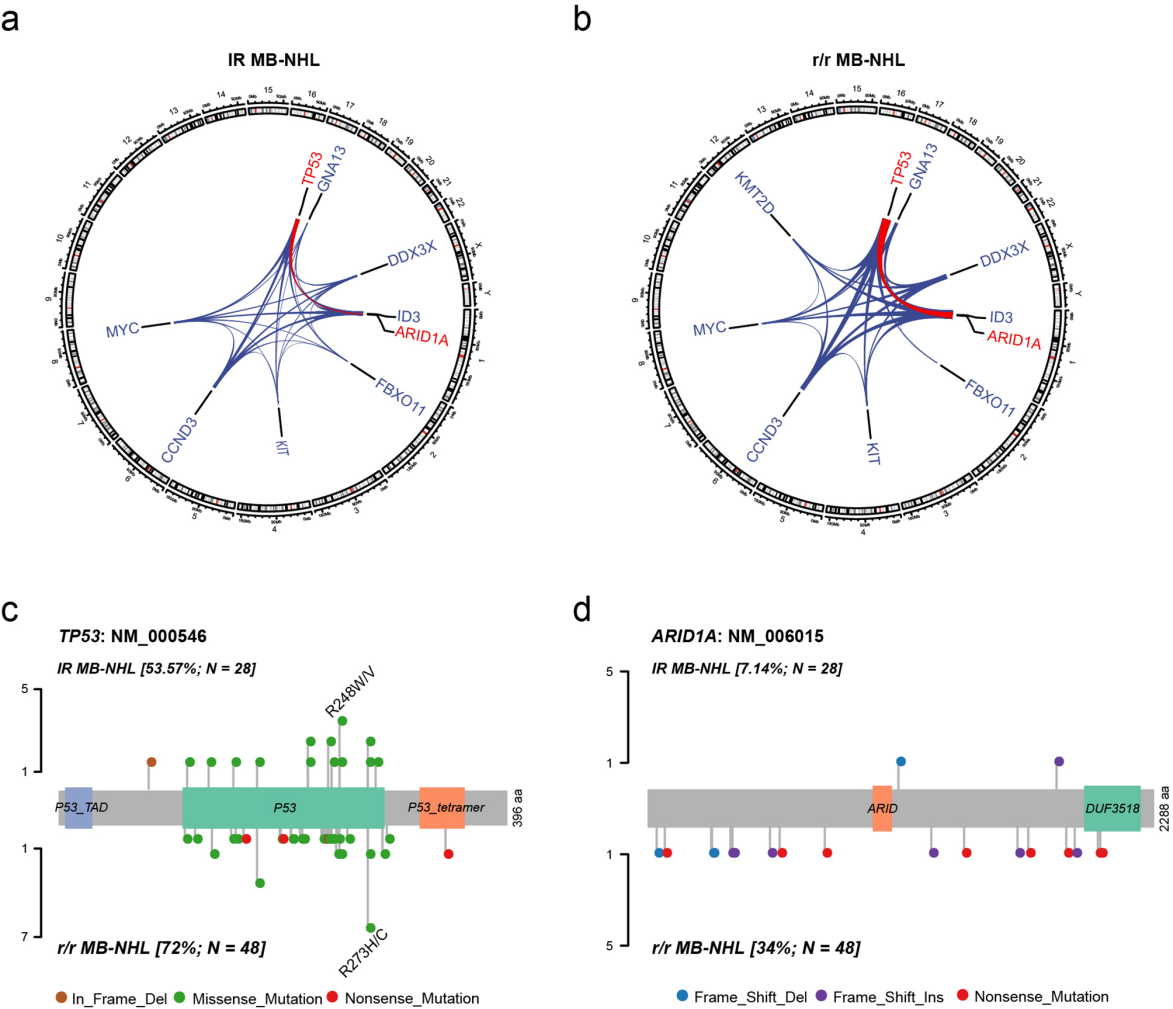
Gene mutations of *TP53* and *ARID1A* in r/r MB-NHL and initial remission patients. (**a**) Co-mutations between genes in cases with IR Mb-NHL (left) and (**b**) r/r MB-NHL (right). The red lines indicate co-mutations of *TP53* and *ARID1A.* (**c**) The location of amino acid changes resulting from *TP53* and (**d**) *ARID1A* mutations. The upper part indicates IR MB-NHL and the lower part indicates r/r MB-NHL patients. The length of the line indicates the number of mutations. R248 W/V (**c**) is a hot spot mutation in *TP53* in initial remission, and R273H/C (c) is a hot spot mutation in *TP53* in r/r MB-NHL

Focusing on the mutation patterns of *TP53* and *ARID1A*, point mutations in the DNA-binding domain (DBD) were the main type for *TP53*. The *TP53* R248 W/V/Q (4 vs. 2) mutation was more common in IR MB-NHL, but the *TP53* R273H/C (8 vs. 3) mutation was more common in r/r MB-NHL patients. Patients with r/r MB-NHL showed a higher prevalence of frameshift and truncating mutations than IR MB-NHL, and these mutation patterns were the major pathogenic mutations found for *ARID1A*, leading to a loss of its function (Fig. 2c and d).

**Fig. 3:**
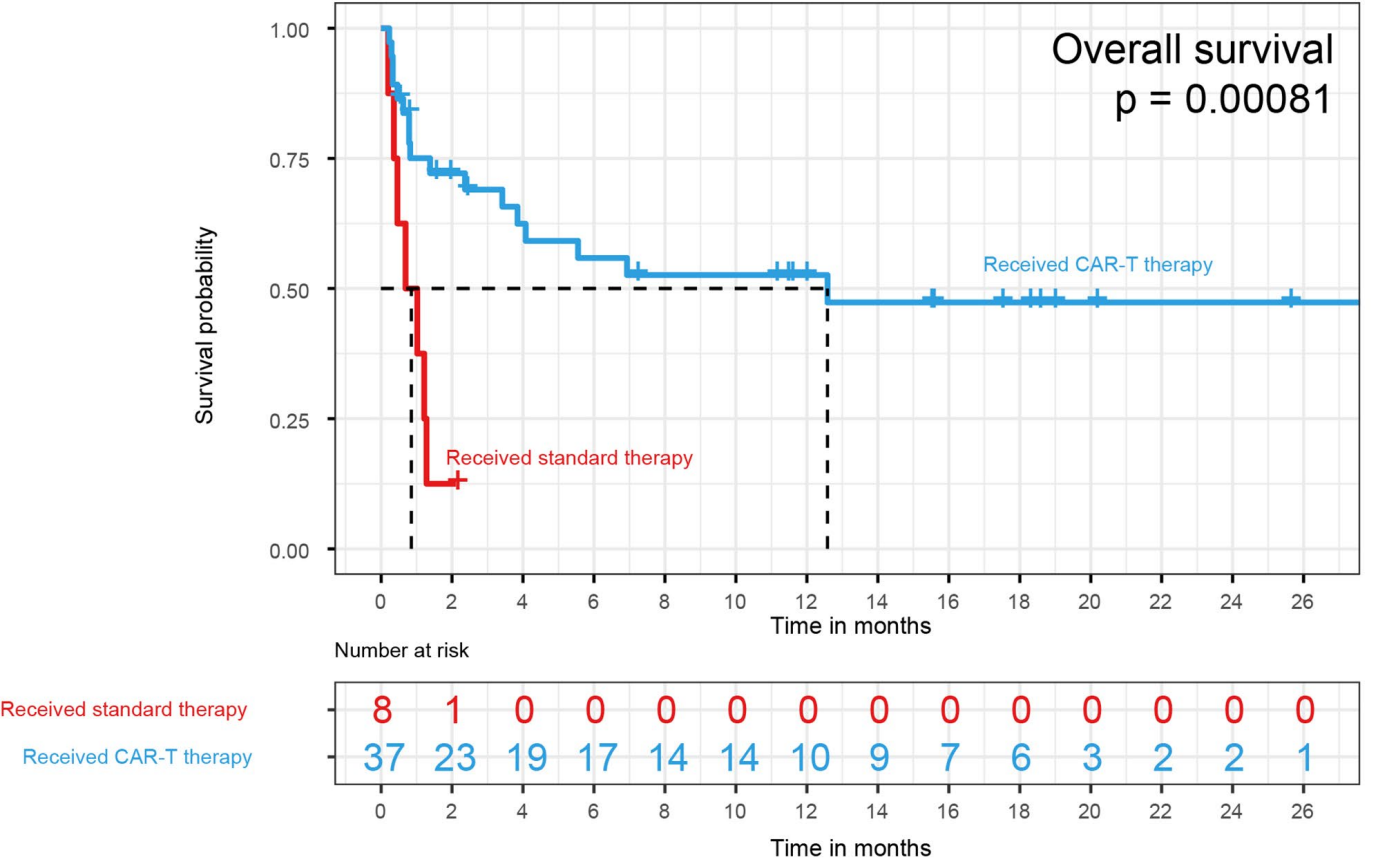
Kaplan‒Meier method comparing overall survival (OS) in r/r MB-NHL between patients who received CAR-T cell therapy (blue; n = 37; mOS = 12.6 months, 95% CI: 3.85-NA months) and those treated without CAR-T cell therapy (red; n = 8). Patients with no survival data were excluded

Further analysis of the VAF showed a significantly higher percentage of VAF for *ARID1A* and *TP53* mutations in patients with r/r MB-NHLs than in patients with IR MB-NHL (*TP53* VAF: IR MB-NHL: median = 20.33%, IQR: 0-47.4%, r/r MB-NHL: median = 53.1%, IQR: 0-83.8%, *P* = 0.0075; *ARID1A* VAF: IR MB-NHL: median = 0%, IQR: 0–0%, r/r MB-NHL: median = 0%, IQR: 0-43.4%, *P* = 0.0025).

### Effects of mutations on the outcome of CAR-T cell therapy

Among the r/r MB-NHL patients, 40 were treated with CAR-T cell therapy. Twenty-two patients (55%) achieved CR and 12 patients (30%) achieved PR, with an Overall response rate (ORR) of 85% (34/40), according to the outcome assessment at the end of each CAR-T cell treatment.

The difference in r/r MB-NHL survival probability between patients treated with and without CAR-T cells was compared (patients with no survival data were excluded). It is worth noting that the median survival in patients treated with CAR-T cell therapy was more than one year (mOS = 12.6 months, 95% CI: 3.85-NA months), with a one-year survival rate of 52.6% and a two-year survival rate of more than 47.3% (Fig. 3). In comparison, patients who did not receive CAR-T cell treatment had poor prognosis, with a median survival of less than one month (*P* = 0.00081). The results showed that CAR-T cell treatment significantly improved the outcome of r/r MB-NHL.

These patients were divided based on the evaluation of the last CAR-T cell treatment into those who responded (CAR-T response patients (CR or PR), 25) and those who did not respond (CAR-T nonresponse patients (NR or PD), 15) to CAR-T cell therapy.

**Fig. 4:**
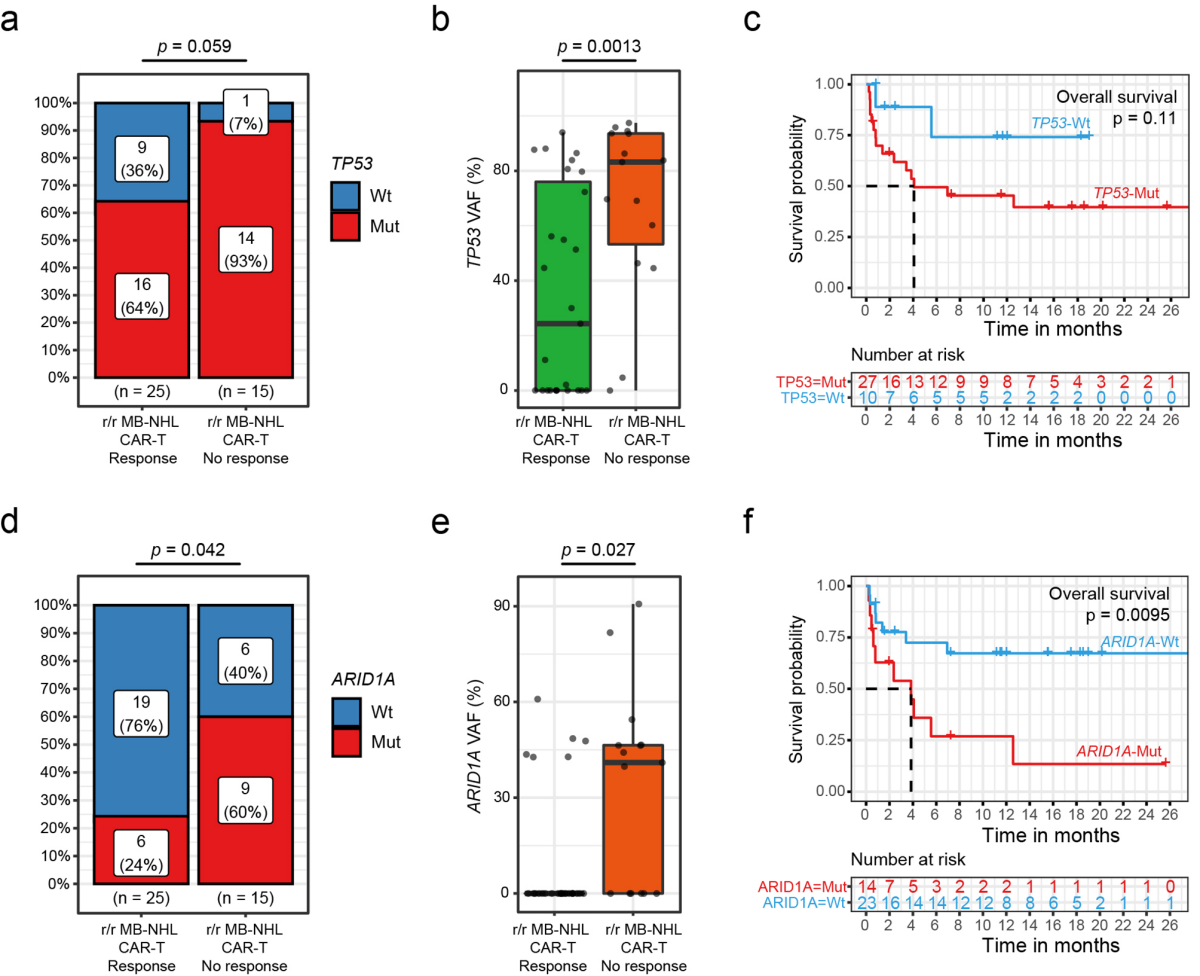
Association of *TP53* and *ARID1A* mutations with the therapeutic effect of CAR-T cell therapy. Comparison of the incidence of (**a**) *TP53* mutations and (**d**) *ARID1A* mutations in the CAR-T cell response group (left) and CAR-T cell no response group (right). Wt, wild-type *ARID1A*; Mut, *ARID1A* mutation. Comparison of variant allele frequencies (VAFs) among (**b**) *TP53* and (**e**) *ARID1A* mutations in the CAR-T cell response group (left, green box) and CAR-T cell no response group (right, orange box). (**c**) Patients with mutations (MUT) in *TP53* (red line) showed worse survival than patients with wild-type (Wt) *TP53* (blue line). (**f**). The survival probability was significantly lower in patients with *ARID1A* mutations (Mut, red line) than in patients with wild-type *ARID1A* (Wt, blue line) (*p* = 0.01)

To further explore molecular markers associated with CAR-T cell treatment, we compared differences in gene mutation profiles between the patients who did and did not respond to CAR-T cell treatment. The results showed that the incidence of *ARID1A* mutation (65% vs. 24%) and the VAF of the gene mutation were significantly higher in patients who did not respond to CAR-T cell therapy than in patients who did respond (*P* = 0.042, Fig. 4d; *P* = 0.027, Fig. 4e, respectively).

Regarding the outcomes of patients treated with CAR-T cell therapy, survival analysis showed that compared with *ARID1A* wild-type patients, patients with *ARID1A* mutations had a poor response to CAR-T therapy and worse DFS (log-rank *P* = 0.0078, Figure S2b) and OS, with a median survival of 3.85 months (95% CI: 0.79-NA month; *ARID1A*-wt, mOS = not reached, log-rank *P* = 0.0095, Fig. 4c). These results show that *ARID1A* status may have an impact on CAR-T therapy.

It was also found that patients who did not respond to CAR-T cell therapy tended to have a higher incidence of *TP53* mutations (93% vs. 54%) than those who did respond (*P* = 0.059, Fig. 4a) as well as a significantly higher VAF of *TP53* (*P* = 0.0013, Fig. 4b). Survival analysis also showed that patients with *TP53* mutations had a shorter median survival of 4.08 months (95% CI: 2.37-NA month) than those without *TP53* mutations (*TP53*-wt, mOS = not reached, log-rank *P* = 0.11, Fig. 4f), which may indicate a trend of poor outcomes, but there was no significant difference in median OS (log-rank *P* = 0.11, Fig. 4f)) or DFS (log-rank *P* = 0.92, Figure S2a) between patients with *TP53* mutations and patients without *TP53* mutations.

### Efficacy of *TP53* and *ARID1A* co-mutation in CAR-T cell treatment

**Fig. 5:**
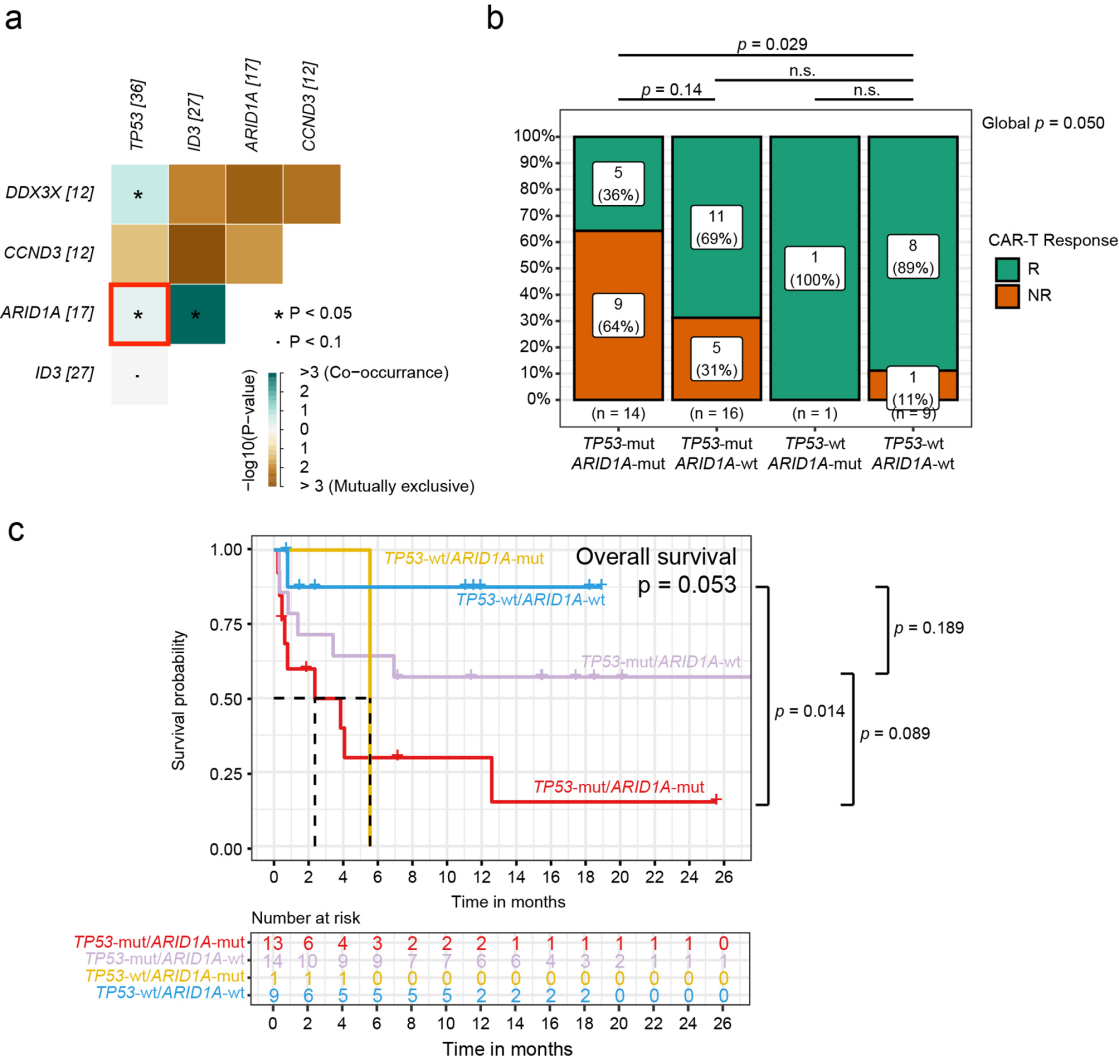
Co-mutations in *TP53* and *ARID1A* are associated with inferior clinical outcomes after CAR-T cell therapy. (**a**) Analysis of co-mutations in r/r MB-NHL. (**b**) Comparison of response rates of CAR-T cell therapy according to TP53 and ARID1A mutation status. (**c**) Survival analyses by Kaplan–Meier according to mutation status in patients treated with CAR-T cell therapy

The above results show that the incidence of co-mutation of *TP53* and *ARID1A* in patients with r/r MB-NHL was significantly higher than that in patients with initial remission (*TP53*-mut&*ARID1A*-mut: n = 16, *TP53*-mut&*ARID1A*-wt: n = 20, *TP53*-wt&*ARID1A*-mut: n = 1, *TP53*-wt&*ARID1A*-wt: n = 11, *P* = 0.018, Fig. 5a). Therefore, we sought to further understand whether *TP53* and *ARID1A* co-mutations have an impact on CAR-T cell therapy. We found that patients (n = 14) with co-mutations of *TP53* and *ARID1A* had a lower response rate to CAR-T cell treatment than the other groups. Only 5 of 14 patients showed a response,the overall response rate was only 36% (Fig. 5b).

Kaplan-Meier survival curves of the effects of *ARID1A* and *TP53* status on OS and DFS showed significantly shorter OS and DFS in patients harboring *ARID1A* and *TP53* co-mutations. (*ARID1A-*mut/*TP53*-mut: mOS = 2.37 months, 95% CI: 0.625 - NA months; *TP53-*wt/*ARID1A*-wt: mOS = not reached, log-rank *p* = 0.014, Fig. 5c) (*ARID1A-*mut/*TP53*-mut: mDFS = 4.06 months, 95% CI: 0.53-NA months; *TP53-*wt/*ARID1A*-wt: mDFS = not reached, log-rank *p* = 0.0039, Figure. S2c).

Therefore, although CAR-T cell treatment significantly improved survival in r/r MB-NHL patients, patients with *ARID1A* and *TP53* co-mutation had poor outcome after treatment when compared to those without such mutations.

## Discussion

The outcome of r/r MB-NHL in children is very poor [6, 8, 24, 25]. Although most patients improve after treatment with CAR-T cells, some patients still experience relapse and rapid progression [26]. We explored a critical clinical question: are there molecular markers that predict the outcome of CAR-T cell therapy in r/r MB-NHL pediatric patients? We analyzed the molecular variation characteristics of MB-NHL in 89 children. To the best of our knowledge, this is the first study to find that *ARID1A* mutations are associated with poor outcome after CAR-T cell therapy in children with r/r MB-NHL. At the same time, a significant finding was that co-mutation of *ARID1A* and *TP53* in Chinese children with r/r MB-NHL is associated with insensitivity to chemotherapy and CAR-T cell treatment.

*ARID1A* is one of the most commonly mutated genes in cancers [27–29]. Expressing a subunit of the SWI/SNF chromatin remodeler, *ARID1A* impacts transcription initiation and elongation [30, 31], participates in control of the PI3K/AKT/mTOR pathway and is associated with *EZH2* methyltransferase activity, steroid receptor modulation and regulation of p53 targets [32–34]. Studies have shown that *ARID1A* has a crucial role in regulating gene expression that drives oncogenesis or tumor suppression and that deletion of *ARID1A* promotes tumor progression. In this study, the incidence of *ARID1A* was higher in r/r MB-NHL patients than in IR patients (35% vs. 7%), indicating that *ARID1A* mutations are enriched in resistant disease and that these patients might be insensitive to initial chemotherapy. A study in ovarian cancer showed that *ARID1A* alterations may also mediate resistance to platinum chemotherapy and estrogen receptor degraders/modulators [35], which further supports our findings that *ARID1A* deficiency may render patients insensitive to initial chemotherapy and be associated with relapse. Moreover, consistent with other studies, most of the *ARID1A* mutations we detected are classified as loss-of-function mutations, which were dispersed throughout the coding sequence [36]. Most of them are truncating or frameshift mutations. These types may lead to disruption of protein functional domains or mediate mRNA degradation, thus disrupting *ARID1A* gene function [37, 38] and leading to tumor cell progression and patient recurrence.

CAR-T cell therapy is a good option for pediatric patients with r/r MB-NHL [9]; however, patients with *ARID1A* mutations have lower rates of response and survival after CAR-T cell therapy. CAR-T cell exhaustion is partly attributed to prolonged exposure to the immunosuppressive microenvironment, upregulation of inhibitory receptors, and persistent CAR stimulation by antigen [39, 40]. There is evidence that *ARID1A* can modulate the tumor immune microenvironment, which underlies its correlation with sensitivity to immunotherapy [40–42]. Julia A. Belk et al. demonstrated through gene editing that *ARID1A* improves T-cell persistence and that anti-*ARID1A* deficiency promotes mutability and potentiates therapeutic antitumor immunity, unleashing tumor immunity in vivo [43]; however, deletion of *ARID1A* gene function and decreased T-cell persistence may be barriers to immune checkpoint blockade and the effectiveness of CAR-T cell immunotherapy [44]. In addition, *ARID1A* mutation is associated with increased expression of PD-L1, which reduces the level of antitumor immune response and may promote immune escape of tumor cells [45, 46]. *ARID1A* is also reportedly closely related to DNA mismatch repair and microsatellite instability [41], which is a possible factor for poor immunotherapy effects caused by *ARID1A* mutation. These conclusions support our view that *ARID1A* mutations are associated with poorer clinical benefit in patients treated with CAR-T cells. Furthermore, *ARID1A* alterations may be a risk factor for insensitivity to chemotherapy or CAR-T cell therapy, and patients should be monitored and regularly followed up.

The negative prognostic impact of *TP53* mutation and its association with drug resistance is well known in many malignancies [47], and some studies have shown that *TP53* aberrations are valuable prognostic markers in CD19-CAR-T cell recipients [18]. Nonetheless, there have been few studies on children with B-NHL. In our study, children with *TP53* mutations showed a trend of adverse outcomes after CAR-T cell therapy, but without reaching a significant level; hence, further research is needed. Multiple studies in solid tumors have demonstrated an inverse relationship between *TP53* and *ARID1A* mutations and even found *ARID1A* and *TP53* mutual exclusivity in ovarian clear cell and uterine endometrioid carcinomas [34, 48]. Remarkably, we found significant co-mutation of *ARID1A* and *TP53* in r/r MB-NHL that was associated with CAR-T cell insensitivity. *ARID1A* has been shown to interact with ligand-bound nuclear hormone receptors and *TP53* through its C-terminal domain and to stimulate the transcriptional activity of these transcription factors [34, 49]. A new study using genetic engineering has shown that *ARID1A* loss probably affects multiple aspects of *TP53*-regulated chromatin and promotes squamous differentiation and acquisition of invasive properties [50]. These may be the causes of the poor prognosis in patients with co-mutation of *ARID1A* and *TP53*, and their molecular mechanisms need to be further studied. In recent years, it has been shown that a high VAF load is associated with poor outcome in patients with hematologic tumors [51] and we also report similar findings.

We have identified molecular abnormalities that may affect the outcome of CAR-T cell therapy in r/r MB-NHL patients, and there is an urgent need to find new solutions. In recent years, with the development of drugs and clinical research, target drugs related to *TP53* or *ARID1A* have been studied in the clinic and laboratory, including combined epigenetic inhibitors to form combined lethal targeted therapies and immune checkpoint inhibitor treatment [52, 53]. CAR-T cell therapy combined with immunosuppressants or cytokine inhibitors has been shown to enhance antitumor efficacy in the treatment of hematologic tumors [54]. In addition, multitarget CAR-T cell combination therapy or radiotherapy combined with CAR-T cell therapy may be more effective than CAR-T cell therapy alone. Our study provides a new clinical basis for further work. In the future, we will continue to pay attention to the follow-up treatment and outcome of lymphoma patients.

This study has several limitations. First, sequencing of tumor samples was driven by clinical decision-making, potentially leading to selection bias. However, we note that population features and outcomes were similar between patients who underwent sequencing and those who did not. Finally, to our knowledge, although this is the first analysis evaluating the role of *ARID1A* in children with r/r MB-NHL treated with CAR-T cells, the sample size was limited. Validation using larger cohorts and prospective trials are warranted to guide CAR-T cell product selection.

## Conclusion

In summary, our study analyzed the genetic features and treatment outcomes of 89 children with MB-NHL, and *ARID1A* mutations are common in pediatric MB-NHL. We demonstrated that children with MB-NHL harboring *ARID1A* or *TP53* and *ARID1A* co-mutation were insensitive to initial conventional chemotherapy and subsequent CAR-T treatment and were associated with disease progression or relapse. Our data suggest that *ARID1A* and *TP53* mutation status should be considered when prognostic factors are evaluated before CAR-T treatment, and this finding would help clinicians customize tailored treatments for their patients.

### Electronic supplementary material

Below is the link to the electronic supplementary material.

Supplementary Material 1

Supplementary Material 2

## Data Availability

The datasets used and/or analyzed during the current study are available from the corresponding author on reasonable request.

## List of abbreviations

MB-NHL: Mature B-cell non-Hodgkin lymphoma
IR: Initial remission
r/r: Refractory or relapsed
CNCL: China Net Childhood Lymphoma
CAR-T cell: Chimeric antigen receptor T cell
EFS: Progression-free survival
OS: Overall survival
DFS: Disease-free survival
CR: Complete response
NR: Partial response
PR: No response
PD: Progressive disease
SNVs: Single-nucleotide variants
InDels: Short insertions and deletions
t-NGS: Targeted next-generation sequencing
PCR: Polymerase chain reaction
VAF: Variant allele frequency
BL: Burkitt Lymphoma
HGBL: High-grade B cell Lymphoma
DLBCL: Diffuse Large B Cell Lymphoma
CNS: Central nervous system
ORR: Overall response rate (ORR)
