# Supplementary material for: Impact of ARID1A and TP53 mutations in pediatric refractory or relapsed mature B-Cell lymphoma treated with CAR-T cell therapy
Source: Cancer Cell Int. 2023 Nov 19;23:281. doi: 10.1186/s12935-023-03122-2 (PMC10657579; doi:10.1186/s12935-023-03122-2)
Supplement: Supplementary file 2 — Supplementary Material 2 [file 12935_2023_3122_MOESM2_ESM.docx]

**Impact of *ARID1A* and *TP53* Mutations in Pediatric Refractory or Relapsed Mature B-Cell Lymphoma Treated with CAR-T-Cell Therapy**

**The supplementary materials include supplemental methods, tables and figures related to the article.**

**1.1 Diagnostic and staging criteria**

(1) Lymphomas were diagnosed according to the World Health Organization Classification of Hematological Malignancies ([1](#_ENREF_1))

(2) International Pediatric Non-Hodgkin Lymphoma Staging System([2](#_ENREF_2))

**Stage I**

Single tumor with exclusion of the mediastinum and abdomen (N; EN; B or S: EN-B, EN-S)

**Stage II**

Single EN tumor with regional node involvement;

≥ Two N areas on same side of diaphragm;

Primary GI tract tumor (usually in ileocecal area), involvement of associated mesenteric nodes, that is completely resectable (if malignant ascites or extension of tumor to adjacent organs, it should be regarded as stage III);

**Stage III**

≥ Two EN tumors (including EN-B or EN-S) above and/or below the diaphragm;

≥ Two N areas above and below the diaphragm;

Any intrathoracic tumor (mediastinal, hilar, pulmonary, pleural, or thymic);

Intra-abdominal and retroperitoneal disease, including liver, spleen, kidney, and/or ovary localizations, regardless of the degree of resection;

Any paraspinal or epidural tumor, regardless of whether other sites are involved;

Single bone lesion with concomitant involvement of EN and/or nonregional N sites;

**Stage IV**

Any of the above findings with initial involvement of the CNS (stage IV CNS), BM (stage IV BM), or both (stage IV combined) based on conventional methods;

**1.2 Inclusion and exclusion for CAR-T treatment**

We conducted a retrospective analysis of data from pediatric patients (aged 0-18) with relapsed or refractory mature B-cell lymphoma (r/r MB-NHL) treated with CD19 CAR T cells and/or CD20 CAR T cells, and/or CD22 CAR T cells between February 2019 and [September](javascript:;) 2021 at Beijing GoBroad Boren Hospital.

**Inclusion Criteria**

(1) Age ≤18 years;

(2) Histologically confirmed mature B cell lymphoma

Confirmation by independent pathology review before enrollment according to the 2016 WHO Classification of Tumors of Hematopoietic and Lymphoid Tissue; A recent tumor sample had to be submitted whenever possible; if it was not clinically feasible, an archival tumor biopsy from the most recent relapse was accepted. Excisional biopsies were preferred; however, a core needle biopsy was allowed. Lymphoma cells definitively expressed CD19 or more B-cell antigens (CD20/CD22) as determined by immunohistochemical (IHC) staining analysis of tumor biopsies obtained before enrollment.

(3) Staging both at diagnosis and at study entry was performed according to the International Pediatric Non-Hodgkin Lymphoma Staging System (IPNHLSS);

(4) Patients had refractory (never obtaining a CR) or relapsed disease after frontline chemotherapy and had a partial response or stable disease as the best response to at least two cycles of salvage chemotherapy including rituximab, and for whom no other curative treatment options were available;

(5) Patient life expectancy ≥12 weeks;

(6) An Eastern Cooperative Oncology Group performance status of either 0 or 1 at screening;

(7) Adequate bone marrow reserve without transfusions, defined as absolute neutrophil count >1000/mm^3^, absolute lymphocyte count >300/mm^3^, platelets ≥50,000/mm^3^, and hemoglobin>8.0 g/dl;

(8) Adequate organ function

Renal function required serum creatinine of ≤1.5×upper limit of normal (ULN); liver function required alanine aminotransferase ≤10×ULN and bilirubin

≤3× ULN;

(9) Provided written informed consent before any screening procedures;

**Exclusion Criteria**

1. Use of any of the following medications

Therapeutic doses of steroids, which had to be stopped >72 hours before leukapheresis and before CAR T-cell infusion; immunosuppressive medication, which had to be stopped ≥2 weeks before leukapheresis and before CAR T-cell infusion; cytotoxic drugs other than lymphodepleting chemotherapy, which were not to be given within 2 weeks before leukapheresis and within 2 weeks before CAR T-cell infusion; rituximab , which was not used within 4 weeks before infusion central nervous system disease prophylaxis, which had to be stopped >1 week before CAR T-cell infusion; and prior radiation therapy within 2 weeks before CAR T-cell infusion.

(2) Active replication of prior infection with hepatitis B or active hepatitis C (HCV RNA positive);

(3) HIV-positive patients;

(4) Uncontrolled acute life-threatening bacterial, viral, or fungal infection (i.e., blood culture positive ≤72 hours before infusion);

(5) Other types of malignant tumors;

(7) Cardiac arrhythmia not controlled with medical management;

(8) Acute respiratory failure;

(9) Elevated intracranial pressure;

(10) Diffuse intravascular coagulation.

**1.3 Introduction to CAR T-cell therapies**

PBMCs collected from patients or cryopreserved apheresis products were stimulated with magnetic beads coated with anti-CD3/CD28 antibodies (Thermo Fisher Scientific, Waltham, MA, USA) overnight. Transduction with a lentiviral vector was performed at a multiplicity of a 1:10 infection ratio the next day. Transduced cells were cultured in serum-free medium (Lonza) with 300 IU/ml interleukin-2 for 5-8 days. Failure to manufacture CAR T cells was defined as the harvest of less than 0.1×10^5^ CAR T cells/kg. CD19 and/or CD20 CAR T cells and/or CD22 CAR T cells were evaluated by FCM with proprietary specific CD19 or CD22 CAR T-cell detection reagents (CD19-CAR-Green, CD20-CAR-Green and CD22-CAR-Green, respectively from Shanghai YaKe Biotechnology Ltd., Shanghai, China). CAR T-cell infusion on day 0 at a target dose of 2 × 10^6^ cells per kg (range, 0.1 × 10^6^ to 5 × 10^6^ cells per kg)

**1.5** **Evaluation and follow-up of CAR-T-cell therapy**

On day 0, all patients received a single dose infusion of CAR-T cells. All patients underwent bone marrow aspiration, cerebrospinal fluid examination, and radiology studies on day 30 to determine the response and remission status. The CAR-T-cell count, efficacy, and adverse events were evaluated at d3, d7, d11, d15, d20, d30, d45 and d60 after the first B-cell target CAR-T-cell infusion. When the CRS response basically disappeared and peripheral blood cells returned to a safe level, the patient was given the next B-cell target passive CAR-T-cell sequential therapy if the tumor remained, progressed, or developed new tumor sites after the peak number of CAR T cells decreased. After a decline in the peak CAR-T-cell count, if the patient's tumor was in complete response and CAR T cells were negative by flow cytometry but were still present by molecular detection, the patient was treated with the next B-cell target active sequential CAR-T-cell therapy until completion of multitarget CAR-T-cell therapy expressed by the subject's tumor cells. The hospitalization period of each patient was 60 days after cell transfusion, and the follow-up time was 2 years.

**1.6 Targeted next-generation sequencing process and parameters**

Library Construction Illumina Next Generation Sequencing (NGS) gDNA libraries with UDI were prepared using the KAPA Hyper Plus kit (KAPA Biosystems, USA). After the genomic DNA was interrupted to approximately 200 bp by enzyme digestion, end repair, joint ligation and pre-PCR were performed to complete the prelibrary construction. The complete set of biotinylated long oligonucleotide probes provided by Agilent HaloPlex was used to perform sequence capture of 262 genes (all coding exons). The probe pool was hybridized to the gDNA upstream and downstream of each region of interest. Pooled DNA libraries were subjected to 2×150 paired-end sequencing on a NextSeq550 sequencer (Illumina). The average depth of coverage across the targeted regions was 1500×.

Functionally annotated variants were filtered based on the information retrieved from public databases (dbSNP, 1000 Genomes and ESP6500). No novel sequencing data have been included outside public databases. Variant allele frequency (VAF) was calculated as the number of variant reads divided by the total reads. Gene variants were classified as driver mutations based on the widely accepted genetic criteria. Somatic gene mutations, including base substitutions and small (< 200 bp) insertions or deletions (indels) and splicing, were all included as driver mutations.


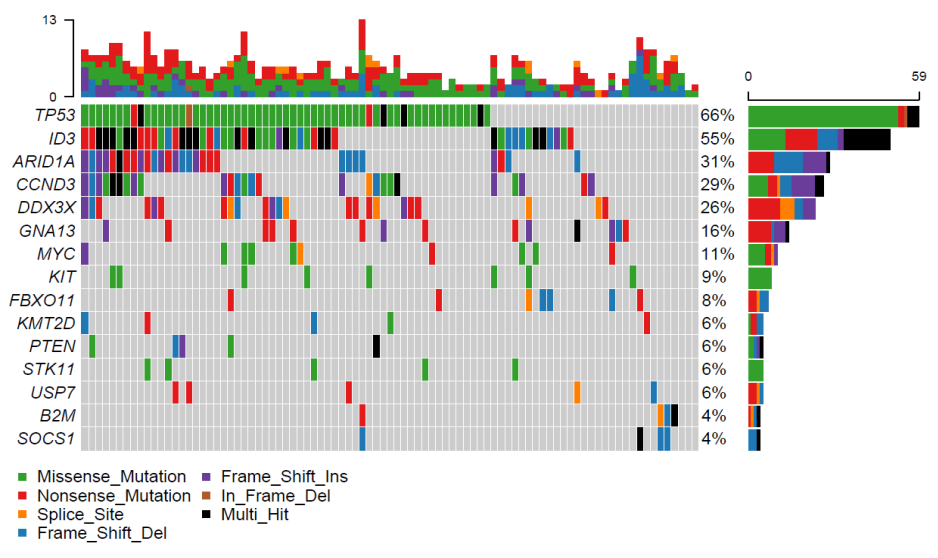


**Figure S1. Gene mutation profile of individual patients (n=89).** Overview of the top 15 mutated genes with different forms of mutations and their frequencies. Side bar plots indicate the incidence of this gene mutation in 89 patients. Side bar plots indicate the incidence of this gene mutation. Top bar plots indicate the number of mutated genes per participant.

Among the r/r MB-NHL patients, 40 were treated with CAR-T cell therapy. Twenty-two patients (55%) achieved CR. We therefore analyzed the effects of *ARID1A* and *TP53* status on DFS in these patients with CR. Kaplan-Meier survival curves showed that patients with *ARID1A* mutations or co-mutations of *TP53* and *ARID1A* had worse DFS after treatment than those without such mutations (Figure S2).


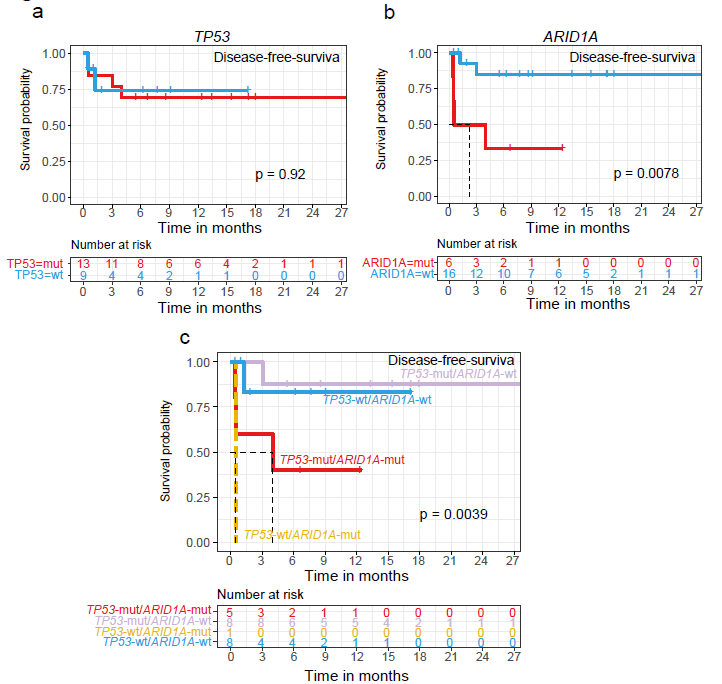


**Figure S2. Disease-free survival by *ARID1A* and *TP53* status in patients treated with CAR-T cell therapies.** (a) DFS by *TP53* status in patients treated with CAR-T cell therapies. (b) DFS by *ARID1A* status in patients treated with CAR-T cell therapies. (c) DFS by Kaplan–Meier according to mutation status in patients treated with CAR-T cell therapy.

**[References](javascript:;)：**

1. Polyatskin IL, Artemyeva AS, Krivolapov YA. [Revised WHO classification of tumors of hematopoietic and lymphoid tissues, 2017 (4th edition):lymphoid tumors]. Arkhiv patologii. 2019;81(3):59-65.

2. Rosolen A, Perkins SL, Pinkerton CR, Guillerman RP, Sandlund JT, Patte C, et al. Revised International Pediatric Non-Hodgkin Lymphoma Staging System. Journal of clinical oncology : official journal of the American Society of Clinical Oncology. 2015;33(18):2112-8.
